# Supplementary material for: Structural basis of Fusobacterium nucleatum adhesin Fap2 interaction with receptors on cancer and immune cells
Source: Nat Commun. 2025 Aug 29;16:8104. doi: 10.1038/s41467-025-63451-w (PMC12397386; doi:10.1038/s41467-025-63451-w)
Supplement: Supplementary file 1 — Supplementary Information [file 41467_2025_63451_MOESM1_ESM.pdf]

Supplementary information for

Structural basis of *Fusobacterium nucleatum* adhesin Fap2 interaction with receptors on cancer and immune cells

Felix Schoepf[1,2], Gian L. Marongiu[1,2], Klaudia Milaj[1,3], Thiemo Sprink[4,5], Judith Kikhney[6], Annette Moter[6] and Daniel Roderer[1,*]

1: Leibniz-Forschungsinstitut für Molekulare Pharmakologie (FMP), Berlin, Robert-Rössle-Straße 10, 13125 Berlin, Germany

2: Freie Universität Berlin, Devision Biology, Chemistry, Pharmacy, 14195 Berlin, Germany

3: Present address: University of Potsdam, Faculty of Science, Karl-Liebknecht-Straße 24-25, 14476 Potsdam OT Golm, Germany

4: Core Facility for Cryo Electron Microscopy of the Charité - Universitätsmedizin Berlin at the Max Delbrück Center, Robert-Rössle-Straße 10, 13125 Berlin, Germany

5: Max Delbrück Center for Molecular Medicine, Technology Platform Cryo-EM, Robert-Rössle-Straße 10, 13125 Berlin, Germany

6: Institute of Medical Microbiology and Virology, University of Leipzig Medical Center, 04103 Leipzig, Germany

*: To whom correspondence should be addressed: roderer@fmp-berlin.de

Supplementary figures.

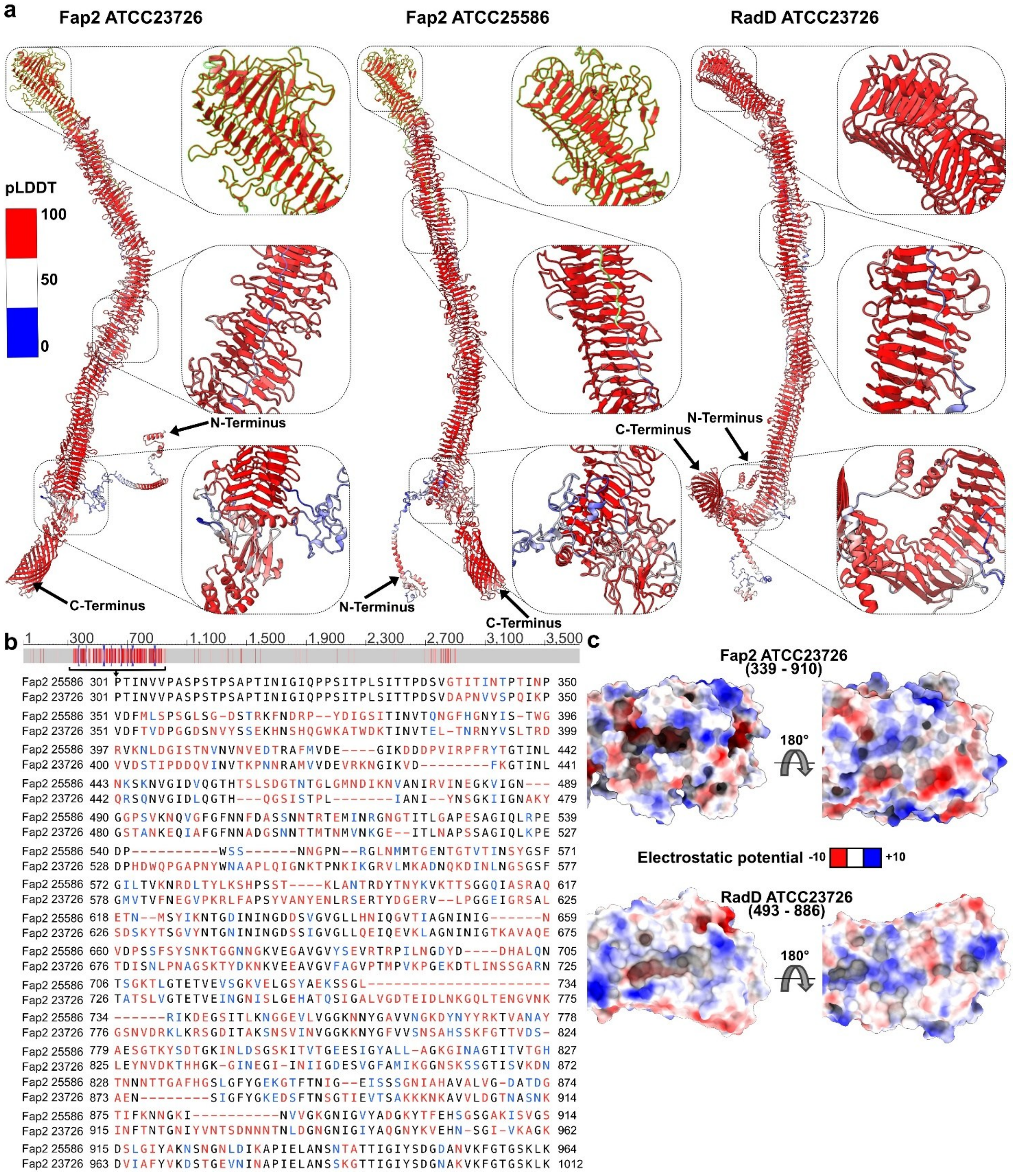

Supplementary Figure 1: AlphaFold2 prediction and sequence alignment of Fap2 and RadD. a) Structure model prediction of adhesins Fap2 from Fn strains ATCC23726 and ATCC25586 and RadD from strain ATCC23726. Models are colored according to prediction reliability (pLDDT). Sections of the matchstick head, the β-helix with the unstructured N-terminus, and the C-terminal end of the β-helix are magnified. The highlighted regions (yellow green frame) correspond to the alignment area magnified in b. b) Sequence alignment of the two Fap2 sequences of *F. nucleatum* ATCC25586 and ATCC23726, focusing on the N-terminal part of the β-helix including a section of

the unstructured region (residues 301-364). The alignment is colored by conservation (black: identical, blue: hydrophilicity/charge conserved, red: not conserved). c) Surface representations of the Fap2 and RadD matchstick regions, colored by electrostatic potential at pH 7.0 (k_BT/e).

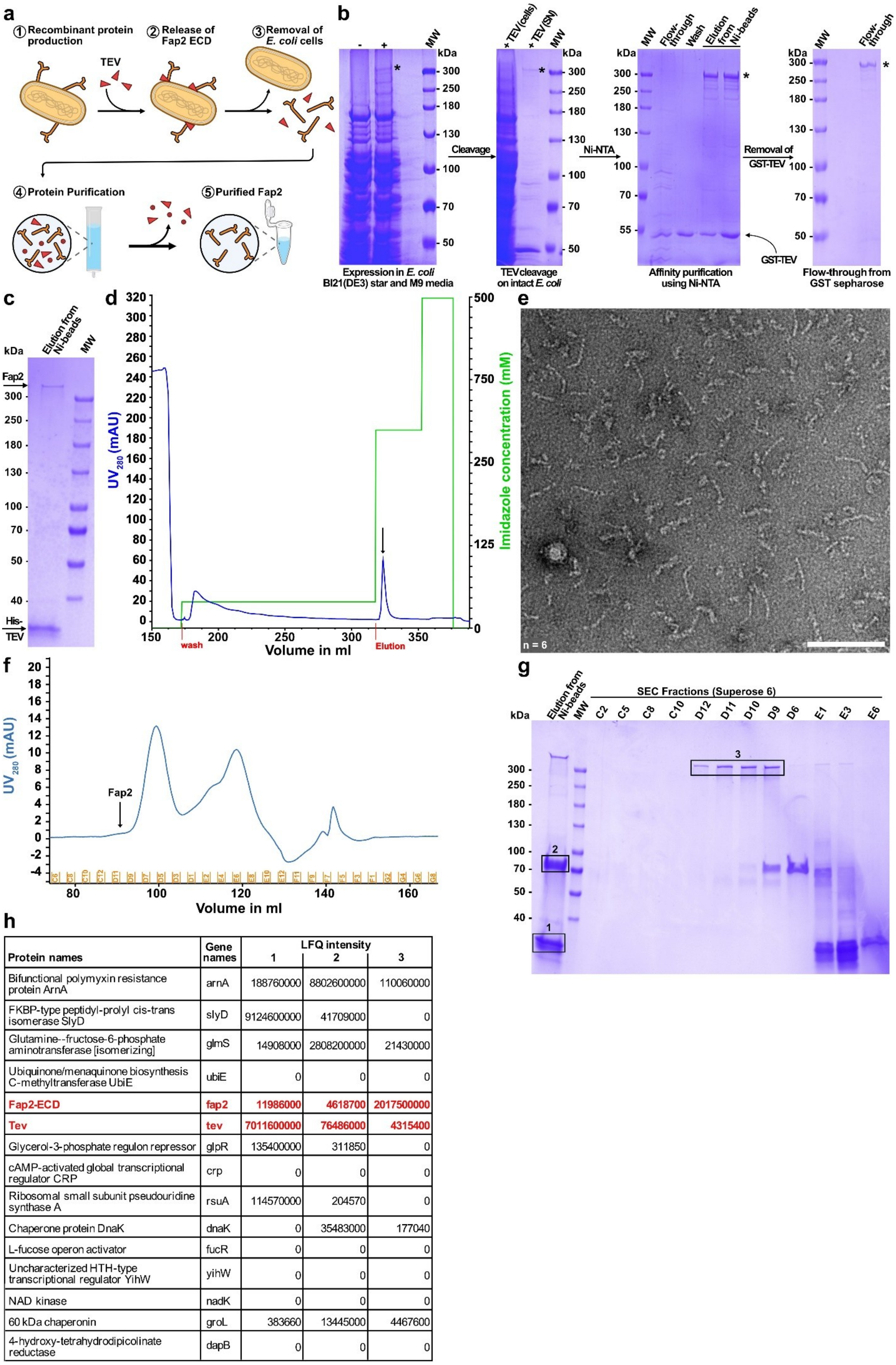

h

Protein names	Gene names	LFQ intensity 1	LFQ intensity 2	LFQ intensity 3
Bifunctional polymyxin resistance protein ArnA	arnA	188760000	8802600000	110060000
FKBP-type peptidyl-prolyl cis-trans isomerase SlyD	slyD	9124600000	41709000	0
Glutamine--fructose-6-phosphate aminotransferase [isomerizing]	glmS	14908000	2808200000	21430000
Ubiquinone/menaquinone biosynthesis C-methyltransferase UbiE	ubiE	0	0	0
**Fap2-ECD**	**fap2**	**11986000**	**4618700**	**2017500000**
**Tev**	**tev**	**7011600000**	**76486000**	**4315400**
Glycerol-3-phosphate regulon repressor	glpR	135400000	311850	0
cAMP-activated global transcriptional regulator CRP	crp	0	0	0
Ribosomal small subunit pseudouridine synthase A	rsuA	114570000	204570	0
Chaperone protein DnaK	dnaK	0	35483000	177040
L-fucose operon activator	fucR	0	0	0
Uncharacterized HTH-type transcriptional regulator YihW	yihW	0	0	0
NAD kinase	nadK	0	0	0
60 kDa chaperonin	groL	383660	13445000	4467600
4-hydroxy-tetrahydrodipicolinate reductase	dapB	0	0	0

Supplementary Figure 2: Purification strategy of Fap2-ECD. a) Schematics of the workflow of Fap2-ECD expression in fusion with the AIDA autotransporter in the *E. coli* outer membrane, followed by release through TEV protease cleavage and purification. b) SDS-PAGEs showing the expression, release by TEV protease, and the first two purification steps. Fap2-ECD is indicated by an asterisk. MW: Molecular weight marker. c) SDS-PAGE with a 4-20% gradient gel of Fap2 after Ni-NTA affinity purification. d) Chromatogram showing the IMAC purification of Fap2-ECD after TEV cleavage. The arrow indicates the main peak of the elution, which has been visualized in e). e) Negative stain EM micrograph of Fap2-ECD after IMAC as indicated in d). Scale bar: 100 nm. The image shows a representative of 6 micrographs with similar results. f) Chromatogram showing size exclusion chromatography (SEC) of Fap2-ECD on Superose 6. g) SDS-PAGE of SEC run in f). Proteins have been concentrated 10x through TCA precipitation before loading on the gel. The three indicated bands were analyzed by peptide fingerprint in h). C2-E6: SEC elution fractions. h) Peptide fingerprint mass spectrometry analysis of the three protein bands indicated in g). Fap2 is the main constituent of sample 3, whereas TEV is the main protein in sample 1. Images of SDS-PAGE gels in (b) and (c) show representatives of at least ten Fap2 purifications with similar results, and the size exclusion analysis shown in (d) was performed in four independent replicates with similar results.

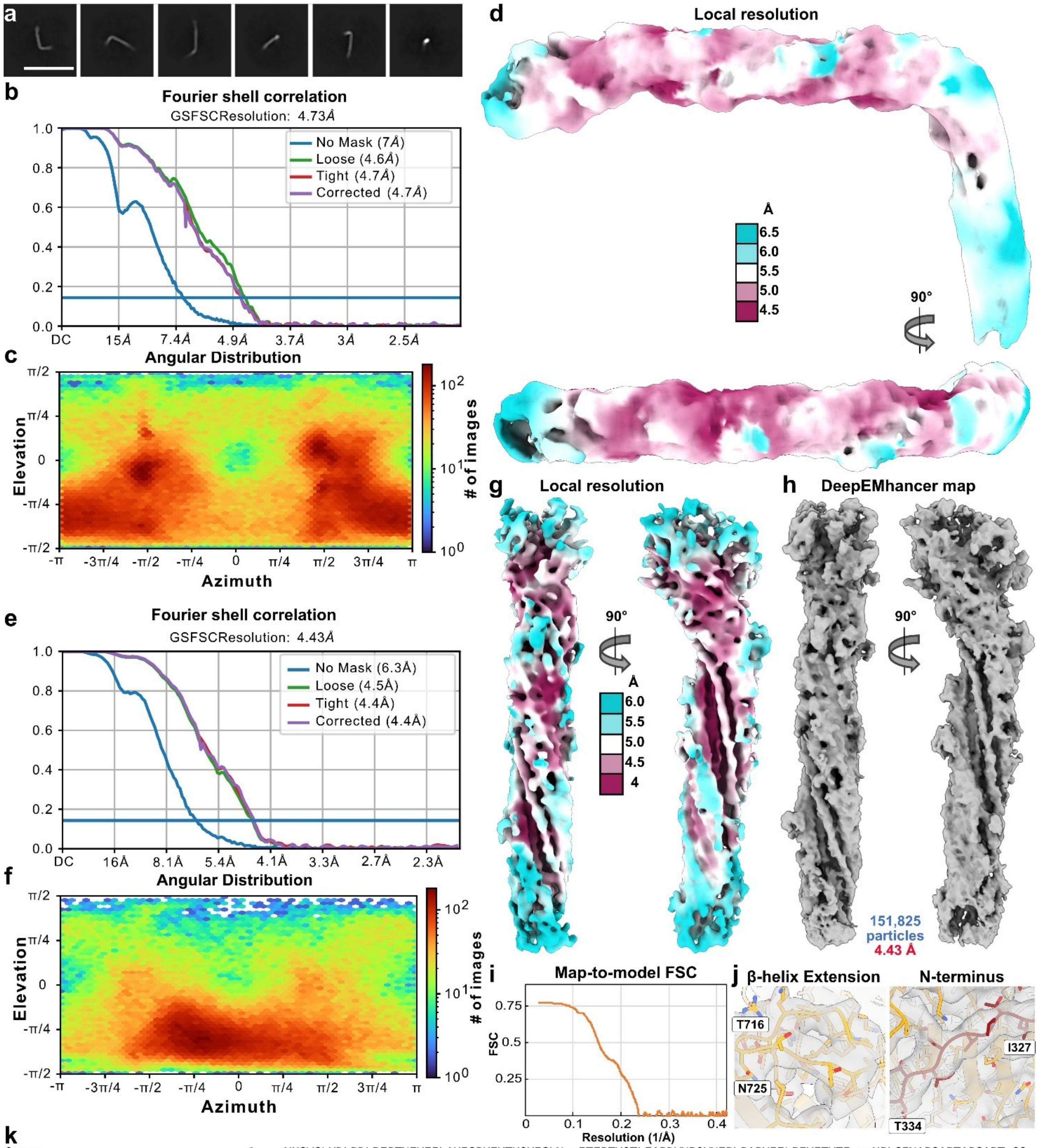

k

```
Query                          1    NKSWGLVDLRDLREPTNEVEILAHISPKEVTKQVISLN--ITEPTVQTLEAPDVNPQVNEPLPAPVIELPEVETVTI---NPLSINAPSAPTAPSAPT 92
F. nucleatum ATCC23726         195  NKSWGLVDLRDLREPTNEVEILAHISPKEVTKQVISLN--ITEPTVQTLEAPDVNPQVNEPLPAPVIELPEVETVTI---NPLSINAPSAPTAPSAPT 286
F. vincentii                   203  NRSWGLVDLKDLKEPTNEVEILARISPKEVTKQAISLN--ITEPTVQTLEAPTVNPQVNEPLPAPVIELPEVETVTI---NPLTINAPSAPTAPSAPT 294
F. nucleatum ATCC25586         195  NKSWGLVDLRDLREPTNEVEILAHISPKEVTKQAISLN--ITEPTVQTLEAPDVNPQVNEPLPAPVIELPEVETVTI---NPLSINAPGAPTAPSAPT 286
F. animalis                    203  NRNWGLVDLKDLREPTNEVEILARISPKEVTKQAISLN--ITEPTVQTLEAPAVNPQVNEPLPAPVIELPEVETVTI---NPLTINAPSAPTAPNAPS 294
F. polymorphum                 195  NRSWGLVDLKDLKEPTNEVEILANISPKEVTKQAISLN--ITEPTVQTLEAPDVNPQVNEPLPAPVIDLPEVETVTI---NPLTINAPSAPSAPTAPN 286
Pseudoleptotrichia goodfellowii 200 NRSWGLVDLRNLKEPTSEVEILARISPKEVTKQAITLN--ITQPAINELEAPIVNPQVNKPLDAPVITLPEVEKVEI---SALNITAPSAPTAPTAPG 291
F. periodonticum               197  NRTWGLVDLLNLKEPTNEVEILARISPKEVSKQALALN--ITAPVVQTLEAPVVKPEPNKPIEAPKINLPSIKKVTI---EALSINPPSAPGVIGVPS 288
Leptotrichia wadei             168   QSYGLASLLHVQEPEVEIQIMANVRPKSVSKEEIAINPKIDMP--REVVRPNINLKVTEPITAPNITFPNVKPINIDIKNPV---APSEPKAIGAPN 256

 Query                         93   ---INI---------AITAPSAPVP---------PTINVVPASPSTPSAPTINIGIQPPSITPLSITTPDSVDA--PNVVSPQIKPVDFTVDPGGDSN 168
F. nucleatum ATCC23726         287  ---INI---------AITAPSAPVP---------PTINVVPASPSTPSAPTINIGIQPPSITPLSITTPDSVDA--PNVVSPQIKPVDFTVDPGGDSN 362
F. vincentii                   295  ---INI---------GITAPSAPIP---------PSINVVPASPSTPSAPSINIGVQPPSITPLSITTPDSVDA--PNVVSPQIKPVDFTVDPGGDSN 370
F. nucleatum ATCC25586         287  ---INI---------GITAPSAPSA---------PTINVVPTSPSTPSAPSINIGIQPPRITPLSITTPDSVGT--ITINTPTINPVDFMLSPSG   359
F. animalis                    295  ---ISI---------GINAPSAPVS---------PTISVVPTSPSAPSAPTINIGIQPPSITALSISTPSTVVP--PEVVSPNITPVDFVIDPAGNS  369
F. polymorphum                 287  ---ISI---------GITAPTAPTAPSAPIAPEVPTITVNPSSPETVSAPTININVTIPKISTLDITTPGSVGT--ISVIAPTVTPVDF         362
Pseudoleptotrichia goodfellowii 292 APVINISITPPNAPNALTAPSAPSV---------PNISVTPSAPSVLTAPNININVTAPSINALTIATPPTVVA--PQVVSPQIKPVDFVIDPSGDA  378
F. periodonticum               289  ---IGI---------TIATPSKPVP---------PTVSVSVNEPSAPGAPTINIPVTPPKIVALNIETPPAVSVTPPTV--SAIKPVAFSVDPTIDSN 364
Leptotrichia wadei             257  ---INI---------TLEAP----Q---------VTLGITP-----PKTPNINISVTPPDITPLTIAKPSEVDL--PTVTPPTINPVDFSIDPTGDS  324
```

Supplementary Figure 3: Cryo-EM and SPA of Fap2-ECD. a) Representative 2D class averages, showing Fap2 in side view and front view. Scale bar: 50 nm. Note that a representative micrograph

is shown in Fig. 1f. b-d) Fourier shell correlation (b), angular distribution heatmap (c), and density map colored by local resolution (d) of the final cryo-EM density of the full Fap2-ECD. e-h) Fourier shell correlation (e), angular distribution heatmap (f), density map colored by local resolution (g), and final density map sharpened with DeepEMhancer (h) of the locally refined longer part of Fap2-ECD including the matchstick region. i) Map-to-model Fourier shell correlation of the locally refined membrane-distal part of the Fap2-ECD including the matchstick region. j) Details of model fitted into the cryo-EM map of the membrane-distal part of the Fap2-ECD. k) Sequence alignment of the N-terminus of Fap2 (residues 195 - 362) from Fn ATCC23726 with related autotransporter sequences from other *Fusobacteriota*.

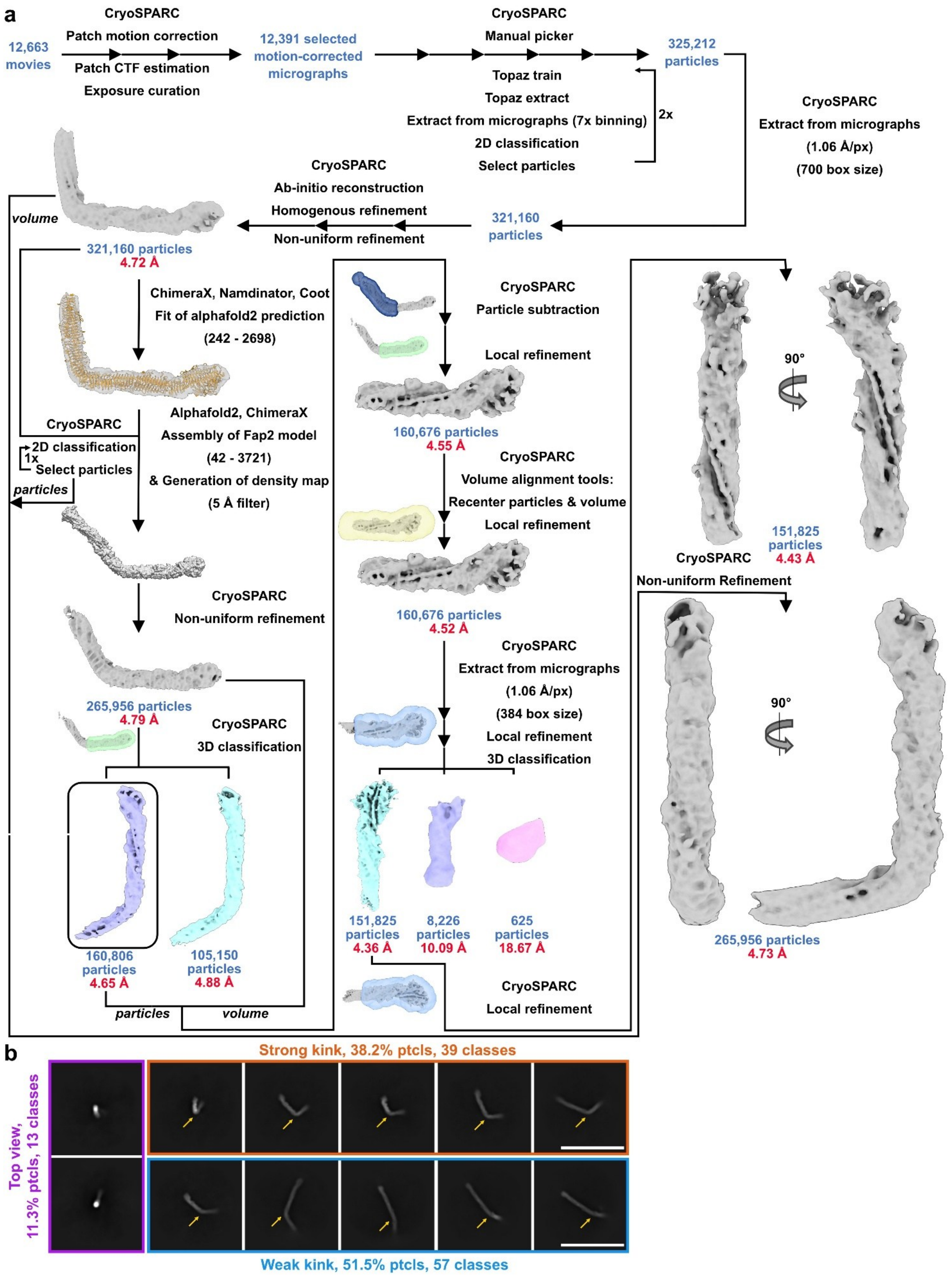

Supplementary Figure 4: a) SPA data processing scheme of the Fap2-ECD. Masks for signal subtraction or local refinements are shown transparent. Note an unconventional data processing step that included the verification of the resulting Fap2-ECD map (full-length, left side) with a map

generated from an AlphaFold2 model. This is required because of the unique shape of Fap2 – slightly tilted orientations of side views could otherwise be interpreted as full side views and result in shortened reconstructions. This was occurring in heterogeneous refinements in CryoSPARC, which were therefore not part of the final processing pipeline. b) Representative 2D class averages of Fap2-ECD, qualitatively sorted according to their kink angle within the β-helix. Strong kink: angle below 110°, weak kink: angle above 110°. Scale bar: 50 nm.

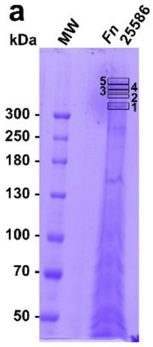

b

Protein names	Gene names	LFQ intensity 1	LFQ intensity 2	LFQ intensity 3	LFQ intensity 4	LFQ intensity 5
Haloalkane dehalogenase	dhaA	0	0	6663100000	28724000	0
Methylated-DNA-protein-cysteine methyltransferase	MGMT	0	0	4622000000	0	0
**RadD**	**RadD**	**12523000000**	**271020000**	**45087000**	**191520000**	**93909000**
Trigger factor	tig	270220000	227600000	75328000	720420000	363890000
Dermcidin;Survival-promoting peptide;DCD-1	DCD	0	0	0	0	4074100
Ubiquitin related	Ubiquitin related	0	30197000	8575000	71303000	37108000
**Fap2**	**Fap2**	**293800000**	**205270000**	**51167000**	**824620000**	**375500000**
Glutathione-S-Transferase	GST 26	0	9299400	0	0	0
Cystatin-A;Cystatin-A, N-terminally processed	CSTA	0	0	6059600	0	0
Transthyretin	TTR	0	0	41707000	0	0
Ubiquitin related modifying proteins	Ubiquitin related	0	0	0	0	2436800
Calmodulin-like protein 5	CALML5	0	8275700	5953900	0	5531100
Chaperone protein DnaK	dnaK	4666300	3624900	158540000	12469000	5716800
Basic salivary proline-rich protein 1;Proline-rich peptide II-2;Basic peptide IB-6	PRB1	0	22626000	2194600	1190400	0
BPI fold-containing family A member 1	BPIFA1	0	7847200	0	0	0

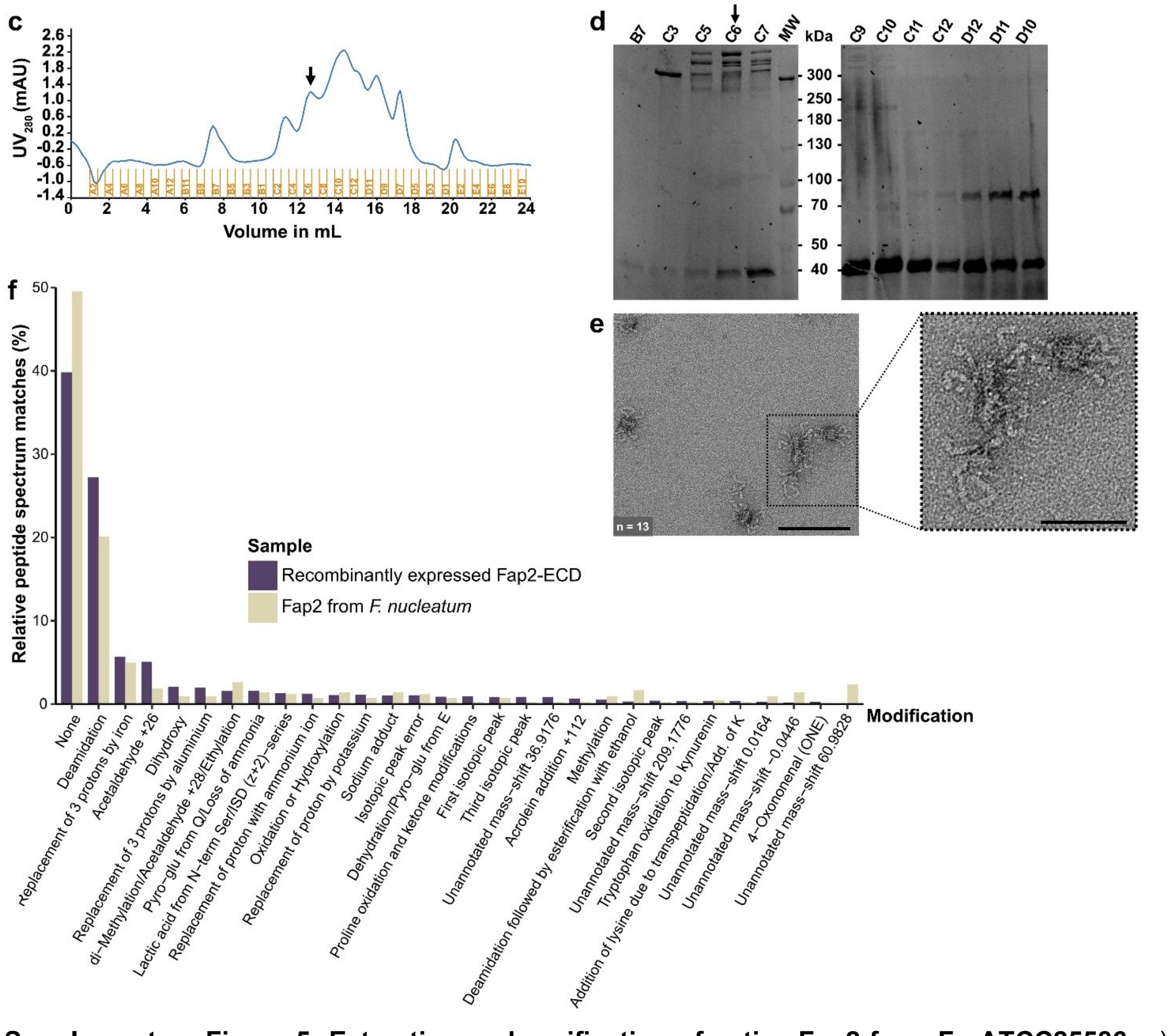

Supplementary Figure 5: Extraction and purification of native Fap2 from Fn ATCC25586. a) SDS-PAGE showing total protein content of Fn ATCC25586. The five indicated bands were analyzed by peptide fingerprint in (b). MW: Molecular weight marker. The image shows a representative of three experiments with similar results. b) Peptide fingerprint mass spectrometry analysis of the five protein bands shown in (a). Fap2 is the main protein in samples 4 and 5, whereas RadD is the main protein in sample 1. Both proteins are nearly equally present in samples 2 and 3. c,d) Chromatogram (c) and SDS-PAGE (d) showing SEC of extracted OM proteins of Fn on Superose 6. The images show representatives of two replicates with similar results. e) Negative stain EM micrograph of SEC

fraction C6 (indicated by arrow in d). Note the rod-shaped molecules that cluster together. Scale bar: 100 nm. The inset shows a zoomed-in region, scale bar: 50 nm. The image shows a representative of 13 micrographs with similar results. f) Comparison of recombinantly expressed Fap2 from *E. coli* with native Fap2 purified from *F. nucleatum* with respect to posttranslational modifications (PTMs) using peptide fingerprint mass spectrometry. No major PTM differences are evident.

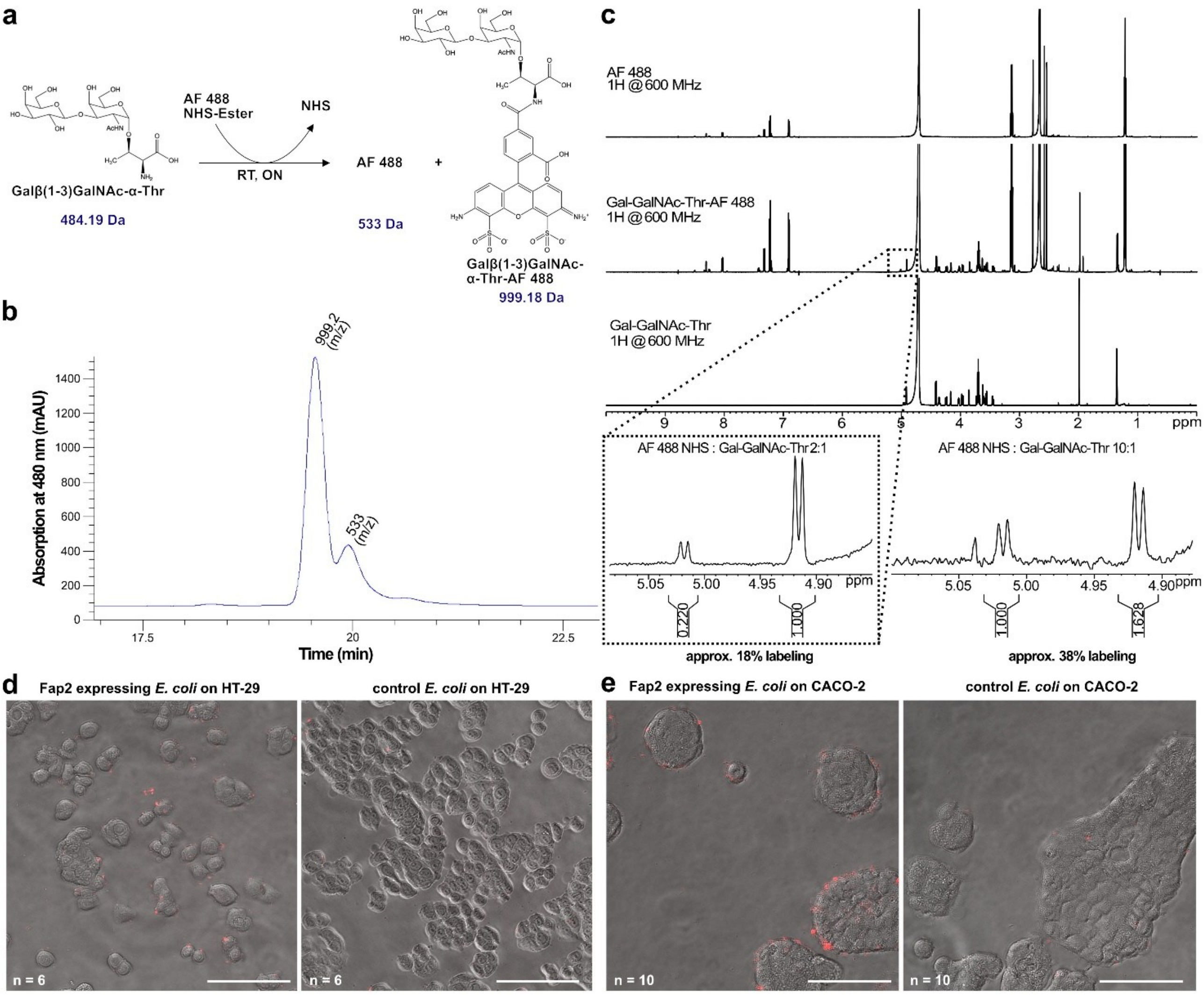

Supplementary Figure 6: Preparation of fluorescently labeled Gal-GalNAc-Thr and CRC cell binding of Fap2-expressing *E. coli*. a) Scheme of the labeling reaction, including the expected masses of the reactants and products. b) Chromatogram showing LC-MS of Gal-GalNAc-Thr after labeling with AF 488 NHS-Ester (Lumiprobe) The mass of the main peak is in agreement with the expected product. c) 1H-NMR spectrum of fluorescently labeled Gal-GalNAc-Thr. The inset shows two characteristic peaks for the educt and product at two ratios of the reactants. d,e) Micrographs that show binding to HT-29 (d) or Caco-2 (e) colon cancer cells of Fap2-expressing *E. coli*. Red channel: *E. coli* labeled with CellBrite Fix 555, gray: bright field image. Scale bars: 50 µm. Images show representatives of 6 (d) and 10 (e) micrographs each with similar results.

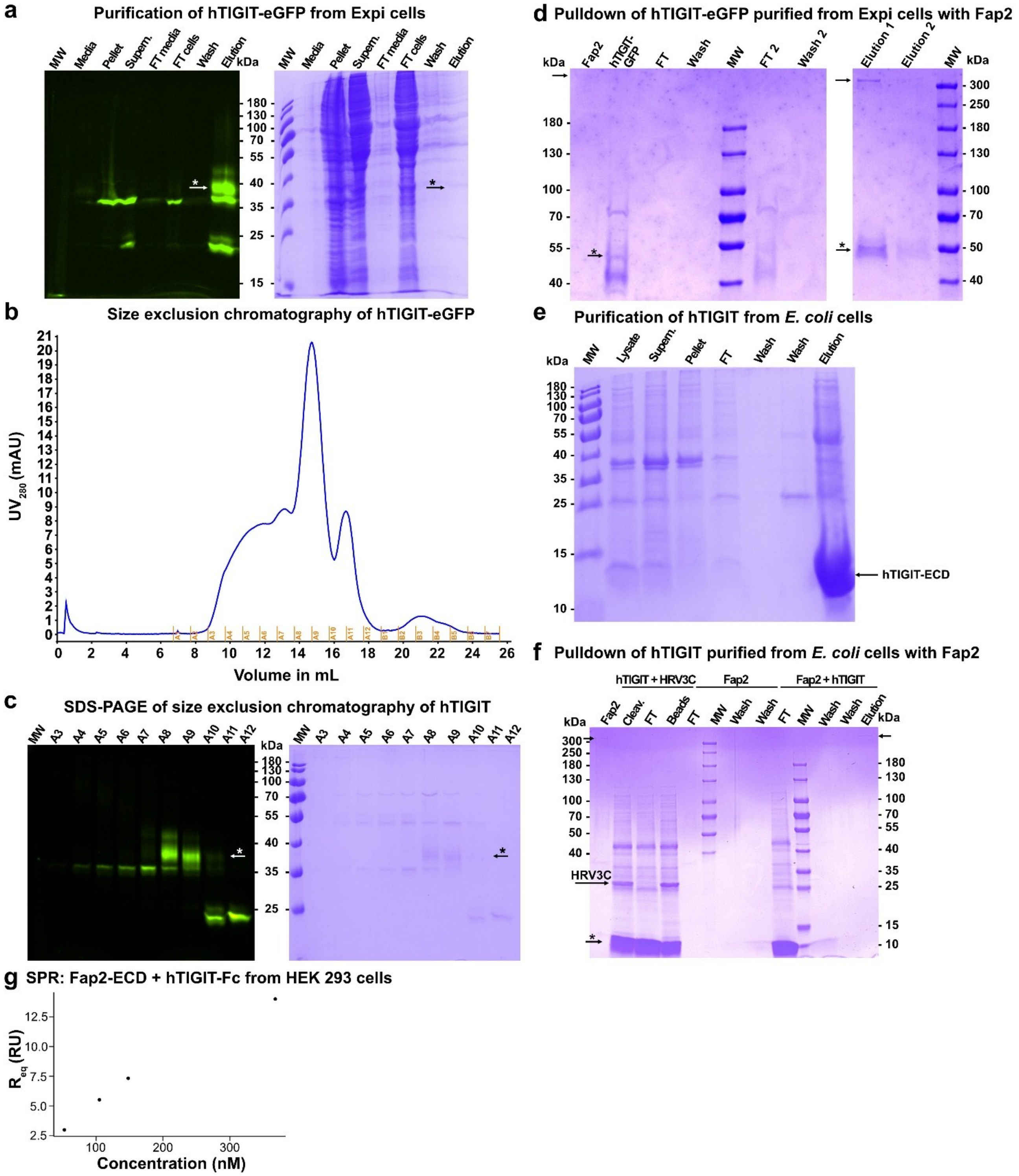

Supplementary Figure 7: Purification of hTIGIT-ECD and pulldown with Fap2. a) SDS-PAGE showing purification of hTIGIT-ECD as fusion protein with eGFP (hTIGIT-ECD-eGFP) from Expi cells. Left: in-gel fluorescence of eGFP, right: coomassie staining. hTIGIT-ECD is indicated (arrow with asterisk). b) Chromatogram showing SEC of hTIGIT-ECD-eGFP as last purification step. c) SDS-PAGE of the SEC in (b). The position of hTIGIT-ECD-eGFP in fractions A8/A9 is indicated. d) SDS-PAGE showing pulldown of hTIGIT-ECD-eGFP (a-c) with Fap2-ECD. The positions of Fap2-ECD and hTIGIT-ECD-eGFP are indicated (bands above 300 kDa and at 50 kDa, respectively). Note that the apparent size differences of hTIGIT-ECD-eGFP in SDS-PAGE originate from the fact that

protein samples were heated in d and not heated in a and c. e) Purification of hTIGIT-ECD that have been insolubly expressed in *E. coli* and refolded, as described by Stengel K.F. et al. (2012). f) SDS-PAGE showing pulldown of hTIGIT-ECD purified from *E. coli* (e) with Fap2. The positions of hTIGIT-ECD (arrow with asterisk) and Fap2-ECD (arrow) are indicated. In contrast to the observations made in (d), no discernible band of hTIGIT is apparent in the pulldown elution fraction. The purifications of hTIGIT-ECD shown in panels (a) – (c) and (e), as well as the pulldown assays with Fap2-ECD presented in panels (d) and (f), were carried out in a single replicate. g) Equilibrium RU values of SPR data of Fap2-ECD binding to hTIGIT-ECD-Fc from Fig. 3d. See also Supplementary Table 3. The limited solubility of Fap2 prevented to use higher concentrations in the experiment.

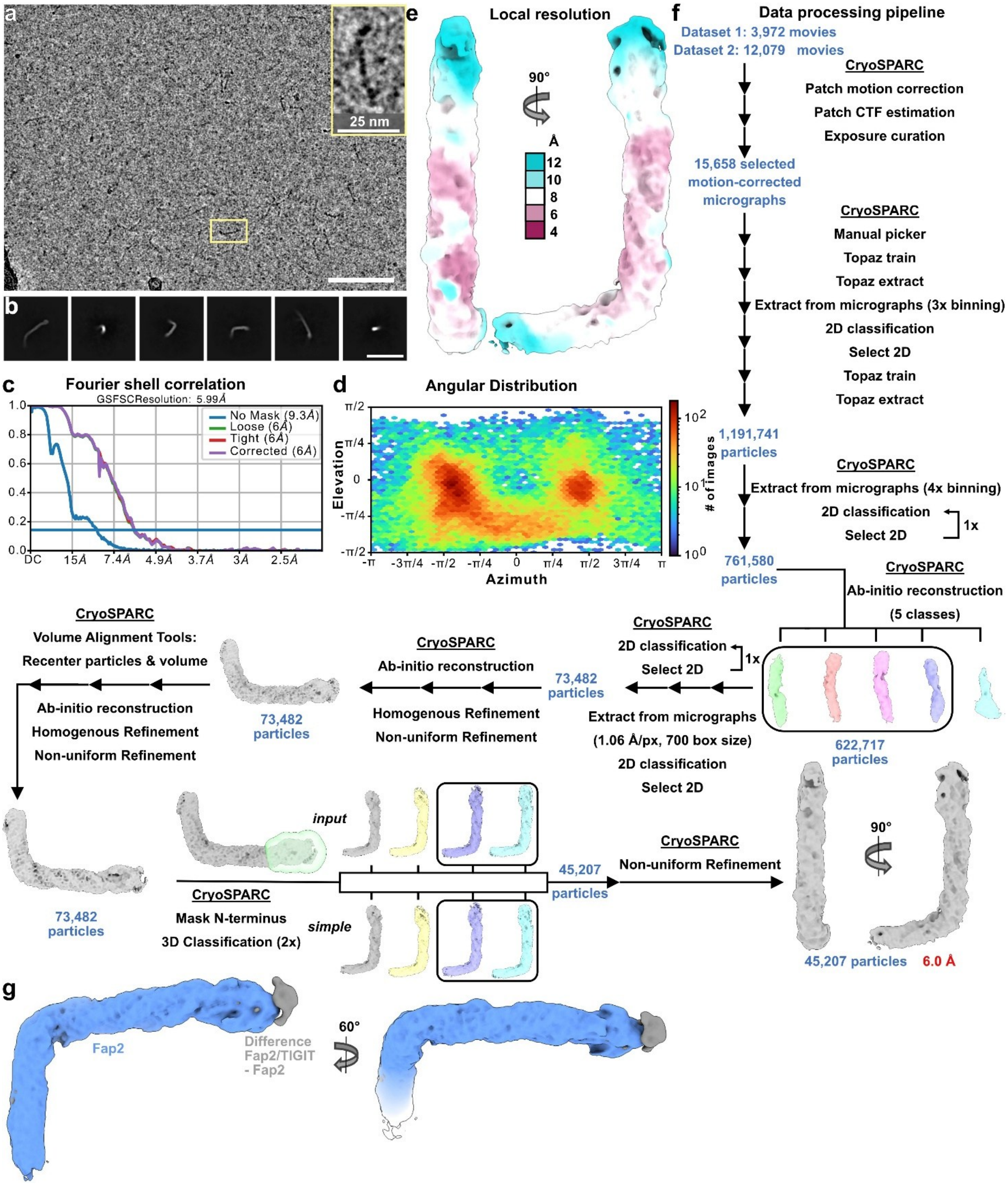

Supplementary Figure 8: Cryo-EM and SPA of Fap2-ECD/hTIGIT-ECD-eGFP complex. a) Cryo-EM micrograph recorded at 300 kV and -2.0 μm defocus. Scale bar: 100 nm. Inset: one molecule in magnified view. The image is a representative of the data set of 15,658 micrographs. b) Representative 2D class averages, showing the complex in side view and front view. Scale bar: 50 nm. c,d,e) Fourier shell correlation (c), angular distribution heatmap (d), and density map colored by local resolution (e) of the final cryo-EM density of the complex. f) SPA data processing scheme of the complex. g) Density maps of Fap2-ECD (blue) and the difference density between Fap2-

ECD/hTIGIT-ECD-eGFP and Fap2-ECD only (gray). Note that no similarly large additional density appears throughout the entire Fap2-ECD than the one corresponding to hTIGIT-ECD at the tip.

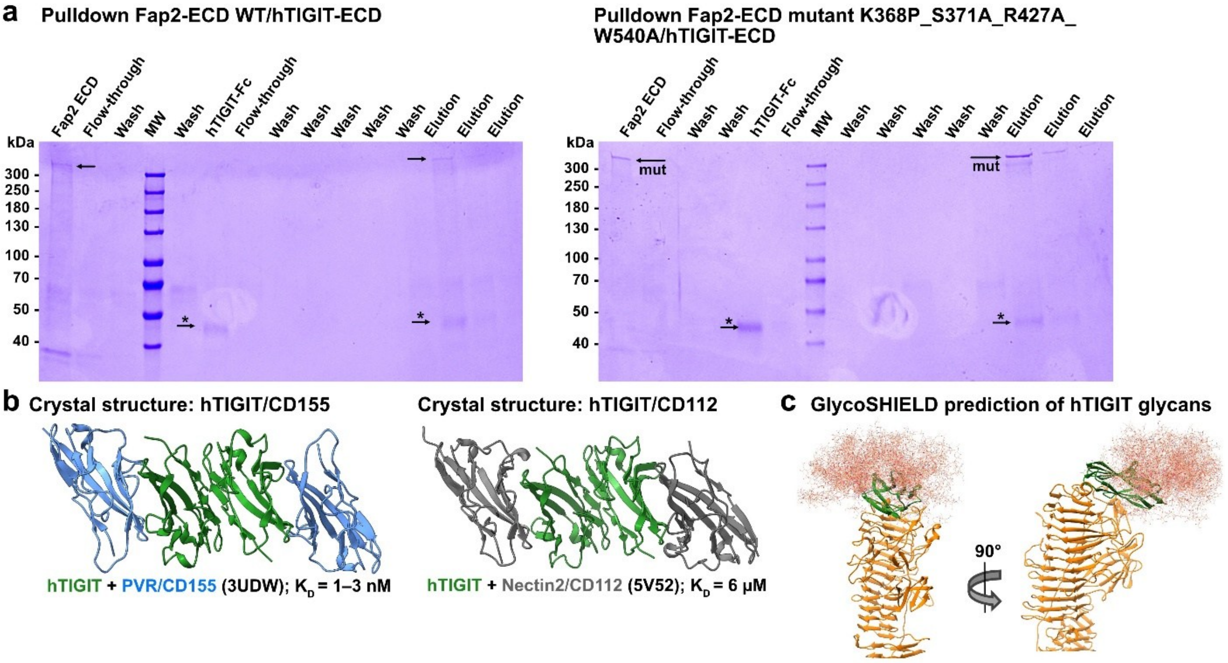

Supplementary Figure 9: Pulldown of Fap2-ECD mutant with hTIGIT-ECD and comparison with co-structures of hTIGIT-ECD with CD155 and CD112. a) Comparison of pulldowns of Fap2-ECD WT with hTIGIT-ECD (left) and Fap2-ECD mutant K368P_S371A_R427A_W540A with hTIGIT-ECD (right). The arrow indicates the position of Fap2-ECD (WT and mutant, respectively), the arrow with asterisk indicates the position of hTIGIT-ECD-Fc. Qualitatively, both Fap2 WT and mutant are able to pull down comparable amounts of hTIGIT. Note that the WT experiment (left) is the same as shown in Fig. 5e. MW: Molecular weight marker. The experiment was repeated twice with similar results. b) Crystal structures of heterotetramers of hTIGIT-ECD with PVR (3UDW) and Nectin2 (5V52). The K_D values of 1-3 nM for hTIGIT/PVR[53] and 6 µM for hTIGIT/CD112[55] are indicated. c) In silico glycosylation of the hTIGIT-ECD model in complex with Fap2. Glycan poses were obtained using GlycoSHIELD[104]. 40 glycan poses (complex biantennal glycan) are shown. This model suggests that glycans are not directly involved in the Fap2/hTIGIT interface.

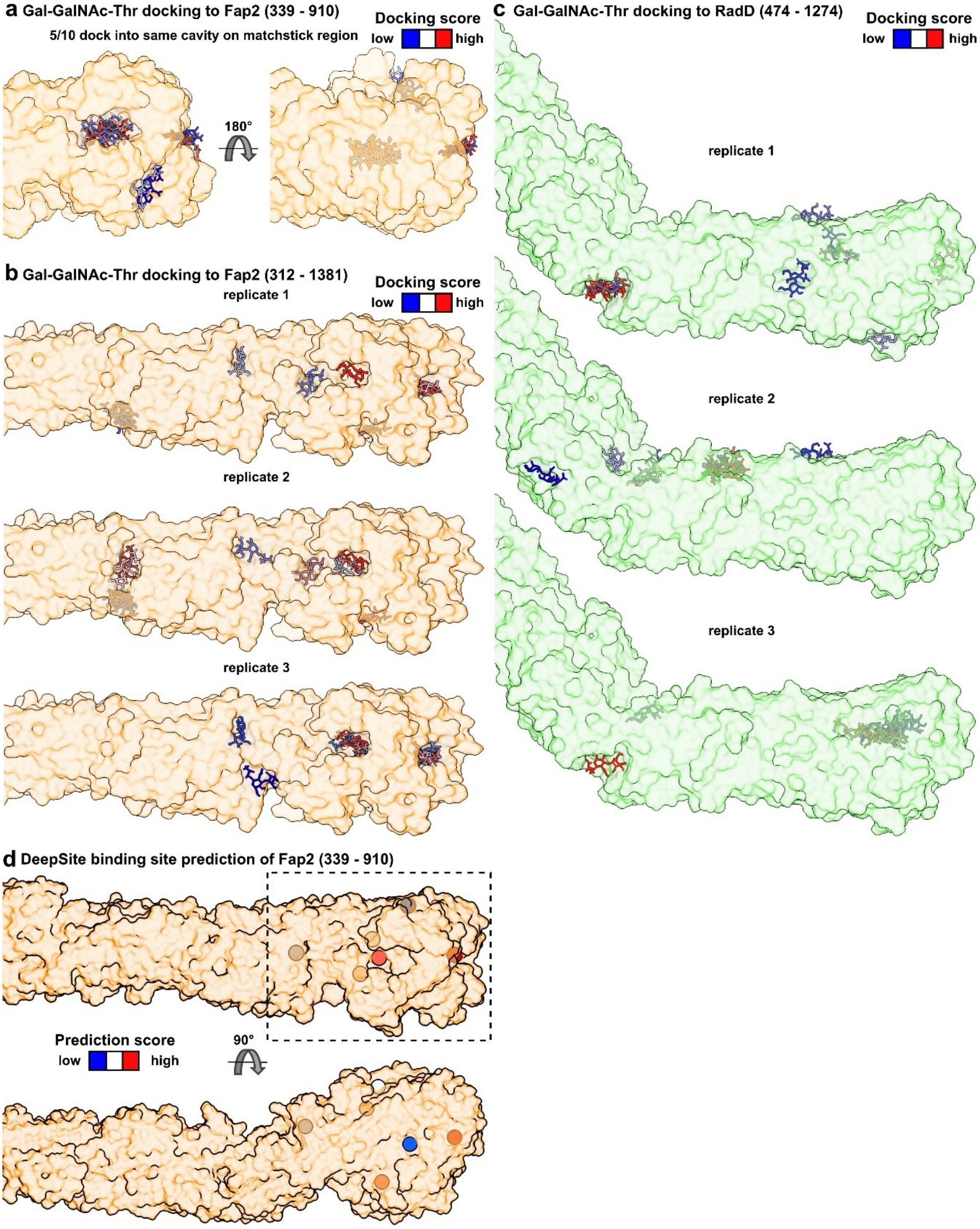

Supplementary Figure 10: Docking of Gal-GalNAc-Thr to Fap2 and RadD. a) Docking of Gal-GalNAc-Thr to the matchstick region of Fap2. 5 out of 10 poses are docked into the proposed binding pit. b,c) Three docking replicates of Gal-GalNAc-Thr to the tips of the β-helices of Fap2 (b) and RadD (c). For Fap2, the docking poses with the highest scores are always docked in the proposed binding pit. For RadD, the docking poses are randomly distributed. The Autodock Vina plugin in UCSF Chimera was used for dockings in (a-c). d) Binding site prediction on the Fap2 matchstick region by DeepSite (Jiménez et al., 2017). The two out of five highest prediction scores (orange-red dots) revealed the proposed binding sites for Gal-GalNAc and hTIGIT.

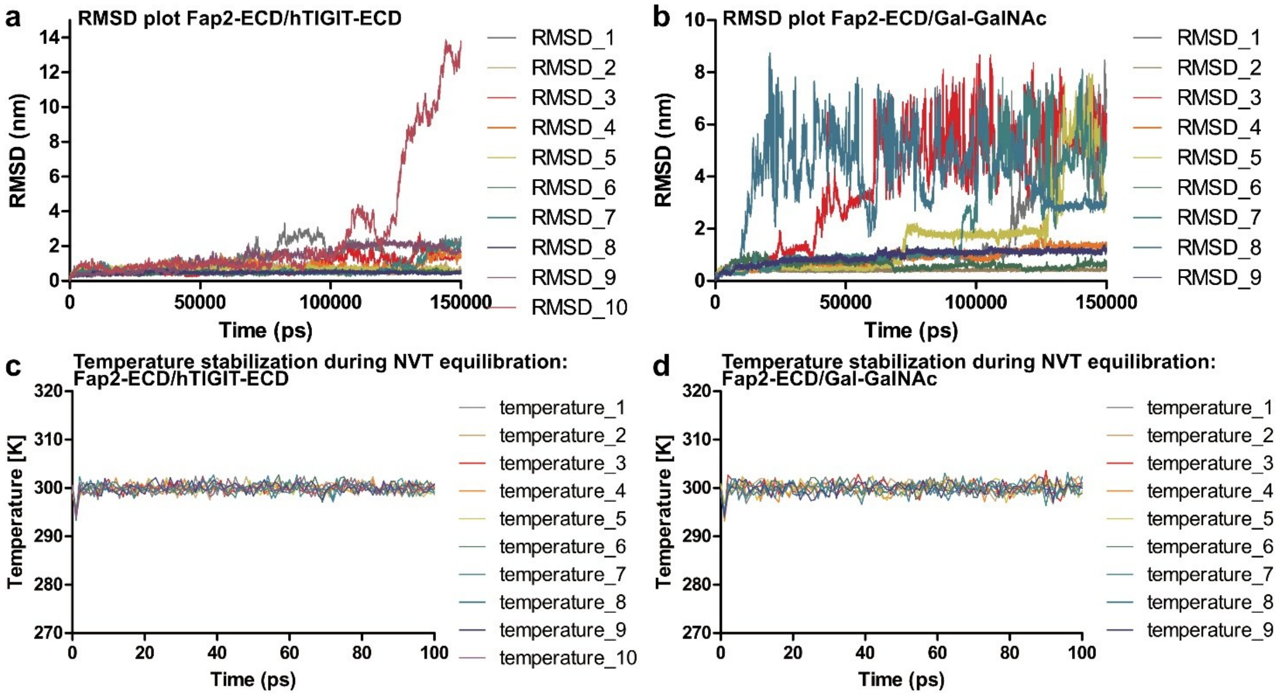

Supplementary Figure 11: RMSD and temperature stabilization plots of MD simulations. a,b) RMSD plots of Fap2-ECD/hTIGIT-ECD (a) and Fap2-ECD/Gal-GalNAc (b) over the simulation times. c,d) Temperature stabilization plots of Fap2-ECD/hTIGIT-ECD (c) and Fap2-ECD/Gal-GalNAc (d). Both systems have been at equilibrium at the start of the production simulation.

Supplementary Tables.

Supplementary Table 1: Cryo-EM data collection, refinement and validation statistics.

	Fap2-ECD (42-3271)<br>EMDB	Fap2-ECD (290-1772)<br>EMDB<br>PDB	Fap2-ECD (42-3271) +<br>hTIGIT-ECD (1-141)<br>EMDB
**Data collection and processing**			
Magnification	81,000		
Voltage (kV)	300		
Camera	Gatan K3 with energy filter		
Electron exposure (e–/Å^2)	59.8		44.6
Defocus range (μm)	-1.5 – -2.6		-1.0 – -2.5
Pixel size (Å)	1.06 (0.53 super resolution)		1.06 (0.53 SR)
Micrographs used	12,391		15,658
Total extracted particle images	375,308		1,191,741
Refined particle images<br>Final particle images	321,160<br>265,956	321,160<br>151,825	761,580<br>45,207
Map resolution (Å)<br>at FSC threshold	4.7<br>0.143	4.4<br>0.143	6.0<br>0.143
Map resolution range (Å)	4.1 – 13.3	3.7 – 7.2	4.6 – 14.1
Map sharpening *B* factor (Å^2)	-192.1	-129.1	-277.1
**Refinement**			
Refinement package	Phenix 1.21-5207		
Model resolution (Å)<br>at FSC threshold		6.5<br>0.5	
Model composition<br>Non-hydrogen atoms<br>Protein residues<br>Ligand<br>Water		 10,826<br>1483<br>0<br>0	
B factors (min/max/mean, Å^2)<br>Protein		 129.25/569.28/235.72	
R.m.s. deviations<br>Bond lengths (Å)<br>Bond angles (°)		 0.003<br>0.815	
Validation<br>MolProbity score<br>Clashscore<br>Poor rotamers (%)		 2.53<br>29.46<br>0.0	
Ramachandran plot<br>Favored (%)<br>Allowed (%)<br>Disallowed (%)		 88.86<br>10.80<br>0.34	

Supplementary Table 2: Comparison of posttranslational modifications on recombinantly purified Fap2-ECD from Fn ATCC23736 and natively purified Fap2 from Fn ATCC25586. See also Supplementary Fig. 5f. PSM: peptide spectrum match.

**Modification**	**Mass Shift**	**Fap2_23726 (*E. coli*) PSMs**	**Fap2_25586 (native) PSMs**	**Relative Fap2_23726 (*E. coli*) PSMs in %**	**Relative Fap2_25586 (native) PSMs in %**
None	0	1656	209	39.82	49.53
Deamidation	0.984	1133	85	27.24	20.14
Replacement of 3 protons by iron	52.911	236	21	5.67	4.98
Acetaldehyde +26	26.015	211	8	5.07	1.90
dihydroxy	31,989.	86	4	2.07	0.95
Replacement of 3 protons by aluminium	23.958	82	4	1.97	0.95
di-Methylation/ Acetaldehyde +28/ Ethylation	28.031	65	11	1.56	2.61
Pyro-glu from Q/Loss of ammonia	-17.026	66	6	1.59	1.42
lactic acid from N-term Ser/ISD (z+2)-series	-15.010	54	5	1.30	1.18
replacement of proton with ammonium ion	17.026	52	3	1.25	0.71
Oxidation or Hydroxylation	15.994	45	6	1.08	1.42
Replacement of proton by potassium	37.955	47	3	1.13	0.71
Sodium adduct	21.981	43	6	1.03	1.42
Isotopic peak error	-1.002	44	5	1.06	1.18
Dehydration/Pyro-glu from E	-18.010	37	3	0.89	0.71
proline oxidation to pyroglutamic acid/ Tryptophan oxidation to oxolactone/aldehyde and ketone modifications	13.979	38	1	0.91	0.24
First isotopic peak	1.002	35	3	0.84	0.71
Third isotopic peak	3.007	36	1	0.87	0.24
Unannotated mass-shift 36.9176	36.918	34	1	0.82	0.24
Acrolein addition +112	112.052	28	1	0.67	0.24
Methylation	14.016	22	4	0.53	0.95
deamidation followed by esterification with ethanol	29.015	13	7	0.31	1.66
Second isotopic peak	2.005	17	1	0.41	0.24
Unannotated mass-shift 209.1776	209.178	16	1	0.38	0.24
tryptophan oxidation to kynurenin	3.995	15	2	0.36	0.47
Addition of lysine due to transpeptidation/Addition of K	128.095	16	1	0.38	0.24
Unannotated mass-shift 0.0164	0.0164	12	4	0.29	0.95
Unannotated mass-shift -0.0446	-0.0446	8	6	0.19	1.42
4-Oxononenal (ONE)	154.099	12	0	0.29	0.00
Unannotated mass-shift 60.9828	60.983	0	10	0.00	2.37

Supplementary Table 3: SPR statistics of hTIGIT-ECD-Fc binding to Fap2-ECD

Sample	Parameters													
**Sample**	**Conc**	**Rmax**	**SE(Rmax)**	**ka**	**SE(ka)**	**kd**	**SE(kd)**	**RI**	**SE(RI)**	**ka (1/Ms)**	**kd (1/s)**	**Rmax (RU)**	**RI (RU)**	**KA (1/M)**
		36.2	1.95	5.29E+04	3.12E+03	0.0308	1.10E-03			5.29e4	0.0308	36.2		1.72e6 5.83
**TIGIT-Fc + 52,5 nM Fap-2**	52.5n							-1.38	0.0777				-1.38	
**TIGIT-Fc + 148 nM Fap-2**	148n							0.243	0.132				0.243	
**TIGIT-Fc + 105 nM Fap-2**	105n							-0.684	0.109				-0.684	
**TIGIT-Fc + 368 nM Fap-2**	368n							5.36	0.245				5.36	

**Sample**	**KD (M)**	**Req (RU)**	**kobs (1/s)**	**Chi2**
	e-7			0.477
**TIGIT-Fc + 52,5 nM Fap-2**		2.99	0.0336	
**TIGIT-Fc + 148 nM Fap-2**		7.33	0.0387	
**TIGIT-Fc + 105 nM Fap-2**		5.52	0.0364	
**TIGIT-Fc + 368 nM Fap-2**		14	0.0503	

	Parameters					
**Sample**	**Inject Start**	**Fit Start**	**Fit End**	**Inject End**	**Fit Start**	**Fit End**
**TIGIT-Fc + 52,5 nM Fap-2**	141	141	285	291	291	314
**TIGIT-Fc + 148 nM Fap-2**	139	141	285	288	291	314
**TIGIT-Fc + 105 nM Fap-2**	139	141	285	288	291	314
**TIGIT-Fc + 368 nM Fap-2**	139	141	285	288	291	314

Supplementary Table 4: Molecular docking of hTIGIT-ECD to Fap2-ECD with HADDOCK2.2

**Parameters**	**Values**
HADDOCK score	-14.6 ± 6.7
Cluster size	9
RMSD from the overall lowest energy structure	0.5 ± 0.4
Van der Waals energy	-97.5 ± 6.1
Electrostatic energy	-181.4 ± 20.8
Desolvation energy	-10.0 ± 1.7
Restraints violation energy	1291.6 ± 53.3
Buried Surface Area	2344.8 ± 89.8

Supplementary Table 5: Occupancy of H-bonds during MD simulation of Fap2-ECD/hTIGIT-ECD complex.

donor	acceptor	rep_1	rep_2	rep_3	rep_4	rep_5	rep_6	rep_7	rep_8	rep_9	rep_10	average occu-pancy	Stan-dard devia-tion
**VAL32-Main-N**	**LYS368-Main-O**	97.04%	98.80%	98.84%	97.64%	98.96%	98.28%	97.84%	78.70%	93.76%	98.16%	95.80%	5.88%
**SER371-Side-OG**	**ALA30-Main-O**	65.35%	99.80%	73.14%	99.88%	99.24%	56.24%	73.86%	80.02%	76.26%	88.53%	81.23%	14.47%
**TRP540-Side-NE1**	**PRO65-Main-O**	93.05%	90.25%	0.04%	3.20%	10.47%	65.11%	95.84%	78.90%	15.15%	92.09%	54.41%	39.60%
**ARG427-Side-NH2**	**GLN22-Side-OE1**	33.41%	48.76%	20.38%	50.48%	39.37%	30.86%	9.47%	38.13%	5.92%	13.99%	29.08%	15.10%
**ARG427-Side-NH1**	**HIS24-Side-NE2**	17.27%	47.68%	16.75%	10.23%	55.56%	3.04%	2.08%	66.43%	40.97%	17.91%	27.79%	21.78%
**TRP53-Main-N**	**ASN541-Side-OD1**	55.80%	45.88%	1.24%	4.20%	0.92%	36.93%	20.02%	16.67%	5.60%	48.84%	23.61%	20.31%
**ASN541-Side-ND2**	**TRP53-Main-O**	22.34%	18.90%	0.16%	1.08%	0.80%	31.29%	26.46%	18.31%	6.35%	39.81%	16.55%	13.26%
**LEU25-Main-N**	**HIS372-Side-ND1**	1.52%	3.56%	4.72%	87.25%	15.75%	0.04%	13.03%	0.68%	0.16%	12.31%	13.90%	25.07%

Supplementary Table 6: Occupancy of H-bonds during MD simulation of Fap2-ECD/Gal-GalNAc-Thr complex.

donor	acceptor	rep_1	rep_2	rep_3	rep_4	rep_5	rep_6	rep_7	rep_8	rep_9	average occupancy	standard deviation
**PHE818-Main-N**	**LIG911-Side-O1**	59.14%	0.17%	40.40%	0.49%	82.63%	0.39%	1.73%	0.77%	98.95%	31.63%	37.64%
**LIG911-Side-O5**	**GLU619-Side-OE1**	46.96%	2.21%	34.23%	9.02%	19.46%	27.38%	1.83%	57.32%	64.29%	29.19%	21.97%
**LIG911-Side-O6**	**GLU619-Side-OE1**	3.08%	2.21%	26.33%	11.85%	1.64%	26.89%	4.35%	53.83%	82.47%	23.63%	26.39%
**LIG911-Side-O6**	**GLU619-Side-OE2**	1.13%	0.33%	36.92%	10.98%	3.00%	24.22%	0.29%	50.73%	84.94%	23.62%	27.59%
**LIG911-Side-O5**	**GLU619-Side-OE2**	3.52%	1.53%	32.22%	7.20%	27.89%	21.91%	3.47%	58.09%	36.22%	21.34%	18.14%
**LYS710-Side-NZ**	**LIG911-Side-O13**	35.66%	1.83%	4.53%	3.16%	16.40%	12.68%	4.61%	0.64%	59.41%	15.44%	18.68%
**ARG613-Main-N**	**LIG911-Side-O1**	0.39%	0.05%	5.40%	0.00%	0.00%	40.55%	0.00%	54.88%	0.00%	11.25%	19.85%
**ARG558-Side-NH2**	**LIG911-Side-O5**	43.76%	0.19%	0.00%	0.00%	9.45%	19.47%	0.08%	24.69%	0.00%	10.85%	14.66%
**LYS710-Side-NZ**	**LIG911-Side-O12**	14.65%	2.51%	8.90%	2.43%	16.98%	4.28%	2.20%	2.27%	38.64%	10.32%	11.34%

Supplementary Table 7: Oligonucleotides used in this study.

**Name and purpose**	**Sequence**
generate pAIDA1_Fap2_ECD_8xHis_TEV_AIDAautotransporter	
Fap2_gibson_fw	5'-AATGCATTTGCAGTCGACGAGGAAGTGAACCTGGAGAACAGCCAGG-3'
Fap2_gibson_rev	5'-GTACAGGTTTTCACCCGCGGACGCACGCAGCGCTTGACCTTCCGG-3'
pAIDA_gibson_fw	5'-CGGGTGAAAACCTGTACTTCCAGGGTGAACAGAAACTGATTAG-3'
pAIDA_gibson_rev	5'-CTCCAGGTTCACTTCCTCGTCGACTGCAAATGCATTTCCGATTGTGG-3'
Fap2_C8xHis_KLD_rev	5'-GATGGTGTGCGCTTCCCGCACGCAGCGCTTGACCTTCCG-3'
Fap2_C8xHis_KLD_fw	5'-ACCATCACCATCACCATTCCGCGGGTGAAAACCTGTACTTCCAGGG-3'
generate GalGalNAc mutant of pAIDA1_Fap2_ECD_8xHis_TEV_AIDAautotransporter	
Fap2_R613P_KLD_fw	5'-CCGGTTCTGCCGGGCGGTGAGATTG-3'
Fap2_R613P_KLD_rev	5'-TTCACCATCATAGGTACGCTCGCTACGCA-3'
Fap2_E619A_KLD_fw	5'-GCGATTGGCCGTAGCGCGCTGAG-3'
Fap2_E619A_KLD_rev	5'-ACCGCCCGGCAGAACAC-3'
Fap2_F818P_KLD_fw	5'-CCGGGTACCACCGTGGATAGCCTGGAGTAC-3'
Fap2_F818P_KLD_rev	5'-CTTGCTGCTGTGCGCGCTGTTG-3'
generate hTIGIT mutant of pAIDA1_Fap2_ECD_8xHis_TEV_AIDAautotransporter	
Fap2_hTIGIT_oligo_mutations_fw	5'-GGTGACAGCAACGTGTATAGCAGCGAAC-3'
Fap2_hTIGIT_oligo_mutations_rev	5'-CAGCGGCGCCGCGTTC-3'
Fap2_K368P_KLD_rev	5'-TTCGCTGCTATACACGTTGCTGTCACC-3'
Fap2_W540A_KLD_fw	5'-GCGAACGCGGCGCCGCTG-3'
generate pAIDA_Fap2_ECD_8xHis_TEV_AIDAautotransporter_S314-K1358	
Fap2_S314_gibson_fw	5'-AATGCATTTGCAGTCGACAGCGCGCCGACCATTAACATC-3'
Fap2_K1358_gibson_rev	5'-GATGGTGTGCGCTTCCCTTTTTCTCAACAATTTCGATGCCCTTGGTG-3'
pAIDA1_SP_rev	5'-GTCGACTGCAAATGCATTTCCGATTG-3'
pAIDA1_His_fw	5'-GGAAGCGCACACCATCACCATC-3'
generate pEG_BacMam_TIGIT_ ECD_GFP_StrepII	
BacMambackbone_gibson_rev	5'-GGTGGCTAGCCGGACCGGGATC-3'
BacMambackbone_gibson_fw	5'-CTGGAAGTTCTGTTTCAGGGTCCCGGTG-3'
TIGIT_gibson_fw	5'-GATCCCGGTCCGGCTAGCCACCATGCGCTGGTGTCTCCTCCTGATCTG-3'
TIGIT_gibson_rev	5'-CTGAAACAGAACTTCCAGTGGAATCTGGAACCTGGCACCGTGC-3'
generate pcDNA3.4_hTIGIT_ECD_TM_GFP_TwinStrep	
pcDNA3.4_gibson_fw	5'-GCAGTTCGAAAAAACTAGTAATTAAAAGCTTGGGAGGGTTCGATCCC-3'
pcDNA3.4_gibson_rev	5'-GAGGAGACACCAGCGCATGGTGGCGGATCCGCGCTAGCTAG-3'
TIGIT_to_pcDNA3.4_gibson_fw	5'-CGCGGATCCGCCACCATGCGCTGGTGTCTCCTC-3'
TIGIT_to_pcDNA3.4_gibson_rev	5'-GAACCCTCCCAAGCTTTTAATTACTAGTTTTTTCGAACTGCG-3'
generate pET19_TIGIT_ECD_6xHis	
TIGIT_to_pET19_gibson_fw	5'-ATGACAGGCACAATAGAAACAACGGGGAAC-3'
TIGIT_to_pET19_gibson_rev	5'-TTCTAGGACCTCCAGGAAGATTCTCCCAGTGT-3'
pET19_gibson_fw	5'-CCTGGAGGTCCTAGAATAATGATCCGGCTGCTAACAAAGCCCG-3'
pET19_gibson_rev	5'-GTTTCTATTGTGCCTGTCATGGGACCCTGAAACAGAACTTCCAGACCG-3'

Supplementary Table 8: MD simulation setup parameters.

	Fap2-ECD/hTIGIT-ECD	Fap2-ECD/GalGalNAc-Thr
**Box volume (nm^3)**	1684.0	488.78
**Box shape**	rectangular/cubic	dodecahedral
**Simulation box dimensions (nm)**	11.897 × 11.897 × 11.897	a = (10.22297, 0, 0)<br>b = (0, 10.22297, 5.11149)<br>c = (0, 5.11149, 7.22874)
**Total number of atoms**	173003	77912
**Total number of water molecules**	54299	23114
**Salt concentration**	9.2 μM	4.8 μM
