# Supplementary material for: Structural basis of Fusobacterium nucleatum adhesin Fap2 interaction with receptors on cancer and immune cells
Source: Nat Commun. 2025 Aug 29;16:8104. doi: 10.1038/s41467-025-63451-w (PMC12397386; doi:10.1038/s41467-025-63451-w)
Supplement: Supplementary file 2 — Description of Additional Supplementary Files [file 41467_2025_63451_MOESM2_ESM.pdf]

Description of Additional Supplementary Files

File Name: Supplementary Movie 1

Description: Structure of the Fap2- ECD. The movie shows a fly-through of longitudinal sections of the density map, highlighting the triangular shaped groove that is complemented by the unstructured N-terminus.

File Name: Supplementary Movie 2

Description: Structure of the Fap2- ECD/hTIGIT-ECD complex. The movie shows a rotation, followed by a fly-through of longitudinal sections of the density map. Finally, the Fap2 and docked hTIGIT-ECD model appear in the map.

File Name: Supplementary Movie 3

Description: Representative MD simulation of the Fap2-ECD/TIGIT-ECD complex. The movie shows a simulation over 150 nsec of the best docking result from HADDOCK. Fap2-ECD is colored orange, hTIGIT-ECD is colored green.

File Name: Supplementary Movie 4

Description: Representative MD simulation of the Fap2-ECD/Gal-GalNAc-Thr complex. The movie shows a simulation over 150 nsec of the best docking pose from Autodock Vina. Gal-GalNAc-Thr is depicted gray, interacting residues of Fap2 are depicted violet.
