# Supplementary material for: Structural basis of Fusobacterium nucleatum adhesin Fap2 interaction with receptors on cancer and immune cells
Source: Nat Commun. 2025 Aug 29;16:8104. doi: 10.1038/s41467-025-63451-w (PMC12397386; doi:10.1038/s41467-025-63451-w)
Supplement: Supplementary file 7 — Reporting Summary [file 41467_2025_63451_MOESM7_ESM.pdf]

Corresponding author(s): Roderer, Daniel

Last updated by author(s): Jul 17, 2025

Reporting Summary

Nature Portfolio wishes to improve the reproducibility of the work that we publish. This form provides structure for consistency and transparency in reporting. For further information on Nature Portfolio policies, see our Editorial Policies and the Editorial Policy Checklist.

Statistics

For all statistical analyses, confirm that the following items are present in the figure legend, table legend, main text, or Methods section.

n/a	Confirmed	
	☒	The exact sample size (*n*) for each experimental group/condition, given as a discrete number and unit of measurement
	☒	A statement on whether measurements were taken from distinct samples or whether the same sample was measured repeatedly
	☒	The statistical test(s) used AND whether they are one- or two-sided<br>*Only common tests should be described solely by name; describe more complex techniques in the Methods section.*
☒		A description of all covariates tested
☒		A description of any assumptions or corrections, such as tests of normality and adjustment for multiple comparisons
	☒	A full description of the statistical parameters including central tendency (e.g. means) or other basic estimates (e.g. regression coefficient) AND variation (e.g. standard deviation) or associated estimates of uncertainty (e.g. confidence intervals)
	☒	For null hypothesis testing, the test statistic (e.g. *F*, *t*, *r*) with confidence intervals, effect sizes, degrees of freedom and *P* value noted<br>*Give P values as exact values whenever suitable.*
☒		For Bayesian analysis, information on the choice of priors and Markov chain Monte Carlo settings
☒		For hierarchical and complex designs, identification of the appropriate level for tests and full reporting of outcomes
☒		Estimates of effect sizes (e.g. Cohen's *d*, Pearson's *r*), indicating how they were calculated

Our web collection on statistics for biologists contains articles on many of the points above.

Software and code

Policy information about availability of computer code

Data collection	Cryo-EM data were recorded using EPU (Thermo Fisher Scientific, version 3.3). Cryo-ET data were recorded using the free software SerialEM (version 4.04). Fluorescence micrographs of Fap2-expressing E. coli on human cell lines were recorded using ZEN black edition (Zeiss, version 14.0.24.201). Surface plasmon resonance data were collected using Biacore 3000 control software (version 3.2). LC-MS of Fap2 PTMs was carried out using Orbitrap Instrument control Software V.4.0.
Data analysis	Cryo-EM single particle analysis was carried out in cryoSPARC (version 4.2.1), Topaz (version 0.2.4), Relion (version 4.0), and DeepEMhancer (version 0.14). Cryo tomograms were reconstructed using etomo in IMOD (version 4.11.24). Quantitative analysis of fluorescence micrographs was carried out using Fiji/ImageJ (version 2.14.0), ggplot (version 3.5.1), the ggstatsplot package (version 0.13.0), and the ggsignif (version 0.6.4) in R (version 4.3.2). Surface plasmon resonance data were evaluated in BIAevaluation (Pharmacia Biosensor, version 4.1.1). MS data were analyzed using FragPipe V. 22.0, and MaxQuant V.1.6.2.6 and 2.0.3.0. Cryo-EM maps and models were fitted using UCSF Chimera version 1.17, UCSF ChimeraX version 1.6-1.7 with ISOLDE, WinCoot version 0.9.8.7, and the Namdinator webserver (accessed 2023-09-28). Refinement of protein models was done with Phenix version 1.21.5207.

For manuscripts utilizing custom algorithms or software that are central to the research but not yet described in published literature, software must be made available to editors and reviewers. We strongly encourage code deposition in a community repository (e.g. GitHub). See the Nature Portfolio guidelines for submitting code & software for further information.

Data

Policy information about availability of data

All manuscripts must include a data availability statement. This statement should provide the following information, where applicable:

- Accession codes, unique identifiers, or web links for publicly available datasets
- A description of any restrictions on data availability
- For clinical datasets or third party data, please ensure that the statement adheres to our policy

Cryo-EM maps have been deposited in the Electron Microscopy Data Bank (EMDB) under accession numbers EMD-53048 [https://www.ebi.ac.uk/pdbe/entry/emdb/EMD-53048] (Fap2-ECD), EMD-53049 [https://www.ebi.ac.uk/pdbe/entry/emdb/EMD-53049] (membrane-distal part of Fap2-ECD), and EMD-53052 [https://www.ebi.ac.uk/pdbe/entry/emdb/EMD-53052] (Fap2-ECD/hTIGIT-ECD complex), respectively. The atomic coordinates of Fap2-ECD (290-1772) have been deposited in the PDB under accession code 9QE7 [https://doi.org/10.2210/pdb9QE7/pdb]. The atomic coordinates of the TIGIT IgV domain with PDB accession code 3UCR [https://doi.org/10.2210/pdb3UCR/pdb] have been used for modeling. The source data underlying Figures 1a,e, 2a,c, 3a,c,d, 4f, 5d,e and Supplementary Figures 2b,c,g,h, 4b, 5a,b,d,f, 7a,c-g, 9a, and 11a-d are provided as a Source Data file. The cryo-EM data sets generated in this work are available from the corresponding author on request.

Research involving human participants, their data, or biological material

Policy information about studies with human participants or human data. See also policy information about sex, gender (identity/presentation), and sexual orientation and race, ethnicity and racism.

Reporting on sex and gender	Not relevant.
Reporting on race, ethnicity, or other socially relevant groupings	Not relevant.
Population characteristics	Not relevant.
Recruitment	Not relevant.
Ethics oversight	Not relevant.

Note that full information on the approval of the study protocol must also be provided in the manuscript.

Field-specific reporting

Please select the one below that is the best fit for your research. If you are not sure, read the appropriate sections before making your selection.

☒ Life sciences ☐ Behavioural & social sciences ☐ Ecological, evolutionary & environmental sciences

For a reference copy of the document with all sections, see nature.com/documents/nr-reporting-summary-flat.pdf

Life sciences study design

All studies must disclose on these points even when the disclosure is negative.

Sample size	No sample size calculation was performed. For SPR data (Fig. 3d), sample size (i.e., different analyte concentrations) was limited by the flow chip design and the solubility of Fap2 (precipitation above ~0.3 mg/ml). The number of micrographs indicated in the figures were used for the quantification of E. coli attachment to HT29 cells and HEK293T cells (Fig. 3b,c; Fig. 4e,f; Fig. 5c,d).
Data exclusions	Cryo-EM SPA data of Fap2-ECD (Fig. 1): Micrographs with a CTF fit resolution worse than 8.8 Å and relative ice thickness above 1.106 as derived from Cryosparc Patch CTF were excluded from further analysis.<br>Cryo-EM SPA data of Fap2-ECD/hTIGIT-ECD (Fig. 4): Micrographs with a CTF fit resolution worse than 6 Å and relative ice thickness above 1.178 as derived from Cryosparc Patch CTF were excluded from further analysis.<br>Analysis of E. coli binding to HT29 cells and HEK293T cells (Fig. 3b,c; Fig. 4e,f; Fig. 5c,d): Micrographs with signal below an area threshold of 100 pixels for cell boundaries in case of HT29 or 50 pixels for hTIGIT-GFP in case of HEK293T were omitted during automated analysis.
Replication	Gal-GalNAc binding to Fap2-expressing E. coli (Fig. 3a): The data points represent technical replicates at every concentration.<br>Fap2-expressing E. coli binding to and HEK293T cells (Fig. 3b,c; Fig. 4e,f; Fig. 5c,d): technical replicates were carried out in different wells of Ibidi plates, and images were taken of all of these wells for quantitative analysis.<br>SPR measurements (Fig. 3d): Several concentrations of Fap2 were evaluated.<br>MD simulations: Replicates were carried out as described in the Methods section.
Randomization	Samples were not randomized. All samples of an experimental setup were processed and analyzed in the same way.
Blinding	Investigators were not blinded during data recording and analysis, because knowledge of the sample properties is required to find the optimal way to process cryo-EM SPA data (e.g., 3D classification with focused mask). To exclude sample bias for the evaluation of Fap2-expressing E. coli binding to HT29 cells and HEK293T cells, the data analysis was carried out in an automated manner.

Reporting for specific materials, systems and methods

We require information from authors about some types of materials, experimental systems and methods used in many studies. Here, indicate whether each material, system or method listed is relevant to your study. If you are not sure if a list item applies to your research, read the appropriate section before selecting a response.

Materials & experimental systems

n/a	Involved in the study
☒	☐ Antibodies
☐	☒ Eukaryotic cell lines
☒	☐ Palaeontology and archaeology
☒	☐ Animals and other organisms
☒	☐ Clinical data
☒	☐ Dual use research of concern
☒	☐ Plants

Methods

n/a	Involved in the study
☒	☐ ChIP-seq
☒	☐ Flow cytometry
☒	☐ MRI-based neuroimaging

Eukaryotic cell lines

Policy information about cell lines and Sex and Gender in Research

Cell line source(s)	HT-29 cells derived from a 44-year-old white female were obtained from ATCC (Cat. No. HTB-38).<br>Caco-2 cells derived from a 72-year-old white male were obtained from ATCC (Cat. No. HTB-37).<br>HEK293T cells were obtained from Merck (Ca. No. 12022001)<br>Expi293F cells were obtained from ThermoFisher Scientific (Cat. No. A14527).<br>Jurkat cells were obtained from Merck (Cat. No. 88042803).
Authentication	Cell lines were not authenticated.
Mycoplasma contamination	Cell lines were not tested for mycoplasma contamination.
Commonly misidentified lines<br>(See ICLAC register)	No commonly misidentified cell lines according to the ICLAC register (version 12, released Jan 16th, 2023) were used.

Plants

Seed stocks	Not relevant.
Novel plant genotypes	Not relevant.
Authentication	Not relevant.
